# Radiomic correlates with overall survival in glioblastoma: amide proton transfer (APT) imaging-based feature analysis and integration of clinical factors

**DOI:** 10.1007/s13246-026-01757-9

**Published:** 2026-07-07

**Authors:** Saima Safdar, Nathaniel Barry, Michael Bynevelt, Adriano Polpo, Suki Gill, Pejman Rowshanfarzad, Martin A. Ebert

**Affiliations:** 1 School of Physics, Mathematics and Computing, The University of Western Australia, Crawley, WA Australia; 2 Neurological Intervention & Imaging Service of Western Australia, Sir Charles Gairdner Hospital, Nedlands, WA 6009 Australia; 3 Department of Radiation Oncology, Sir Charles Gairdner Hospital, Hospital Ave, Nedlands, WA Australia; 4 School of Allied Health, The University of Western Australia, Crawley, WA Australia; 5 Centre for Advanced Technologies in Cancer Research (CATCR), Perth, WA Australia

**Keywords:** Radiomics, Glioblastoma, Amide proton transfer, Magnetic resonance imaging, Overall survival

## Abstract

This study identifies amide proton transfer (APT) imaging radiomic features that correlate with glioblastoma survival and explore the relationship of combined radiomic data and biomarkers such as age and MGMT methylation status to survival prognosis. In a retrospective study of 66 glioblastoma patients (2022–2023), SlicerBrats was used for automatic tumor segmentation and 504 radiomic features were extracted from APT images. A two-step feature reduction process was applied: (1) Pearson correlation reduced the features to 59 by mitigating multicollinearity, and (2) a Cox model with best subset selection identified the optimal features using the lowest Akaike Information Criterion (AIC). The Goodness-of-Fit (GOF) test was used to evaluate model adequacy. Bootstrapping with 1000 iterations was used to compute the confidence interval for the concordance index (C-index). Univariate Cox models assessed individual radiomics, while models with multiple features evaluated radiomics alone, with age, MGMT methylation, and both. Kaplan-Meier and log-rank tests were performed to stratify survival groups. The radiomics-only model showed the highest C-index (0.74), AIC (191.35), and GOF (*p* < 0.005). Adding age maintained the C-index (0.74) but raised AIC to 193.16 (*p* = 0.01). The radiomics + MGMT model achieved a C-index of 0.73 and the lowest AIC (190.15). Kaplan-Meier curves and log-rank tests confirmed all models significantly distinguished survival groups (*p* < 0.05). APT-derived radiomic features, especially from edema and necrosis, correlate with glioblastoma survival time. Adding MGMT methylation improved predictive accuracy, achieving the lowest partial AIC and a C-index of 0.73. Further validation in larger cohorts is recommended.

**Supplementary Information:**

The online version contains supplementary material available at https://doi.org/10.1007/s13246-026-01757-9.

## Introduction

 Glioblastoma (World Health Organization [WHO] grade IV astrocytoma [1]) is the most common primary brain malignancy in adults, with 5-year survival rates around 10% [2] for patients who undergo maximal safe tumour resection, followed by radiotherapy and concomitant and adjuvant temozolomide [3]. Magnetic resonance imaging (MRI) is the primary imaging modality due to its superior soft-tissue contrast [4]. Structural sequences, including T1-weighted imaging (T1w), T1w contrast-enhanced imaging (T1CE), T2-weighted (T2w), and T2-Fluid-attenuated inversion recovery (FLAIR), are commonly used for diagnosis and prognosis. Additionally, advanced MRI sequences, such as diffusion-weighted and perfusion-weighted imaging, are increasingly utilized [5].

APT MRI [6] leverages proton exchange between water and amide groups in proteins, enabling non-invasive detection of high-cellularity brain regions without contrast agents [7]. APT imaging correlates with glioma grade, Ki-67 expression, and cell density, serving as a potential biomarker [8, 9]. APT-weighted imaging shows strong prognostic value for overall (OS) and progression-free survival (PFS), with APTwmean identified as an independent predictor [10–13].

Radiomics involves extracting quantitative features from medical images, revealing disease-specific processes that may be imperceptible to the human eye [14]. This approach has gained significant attention, particularly in analysing tumor heterogeneity through texture features [15]. However, the use of radiomics and machine learning approaches on APT images has attracted minimal investigation. One study differentiated gliomas from metastases in 48 patients, achieving an AUC of 0.836 using a multilayer perceptron and 10-fold cross-validation [16]. Another used SVM to predict IDH mutation status with an AUC of 0.84, though on a small test set (*n* = 10) [17]. Research focusing on application of APT radiomics to glioblastoma-specific cohorts is scarce, underscoring the need for targeted studies to integrate APT radiomics with clinical features for survival prediction.

The objective of this study was to identify radiomic features, extracted from APT images acquired on the first follow-up MRI imaging post-surgical treatment in glioblastoma patients undergoing radiochemotherapy, that demonstrate significant hazard ratios when correlated with patient overall survival. These features were combined with clinical parameters to assess feasibility of a model to predict overall survival in patients with histologically confirmed glioblastoma.

## Materials and methods

### Clinical dataset

This retrospective, protocol-driven study included adult patients diagnosed with glioblastoma (WHO grade 4) between 2022 and 2023, all of whom underwent treatment at Sir Charles Gairdner Hospital (SCGH), Perth, Western Australia. Patients were included if they had histologically confirmed glioblastoma and had undergone all four structural MRI sequences as well as Amino Proton Transfer (APT) imaging at the 3-month post-surgical follow-up. Sixty-six patients (see Table 1) met the inclusion criteria and had complete imaging and clinical data available for analysis. Where available, molecular data such as MGMT methylation status were collected.

**Table 1:** The specific retrospective data acquired for each participant

Source	Data
Pre-operative MRI	T1w
	T1CE
	T2w
	T2-FLAIR
Pathology	Histological characteristics
	MGMT promotor methylation
	IDH-mutation
	TERTp-mutation
Radiotherapy plan	Planning CT
	Anatomical Segmentations (RT structure files)
	Dose Plan
Post-RCx MRI:	T1w
4 week follow up (baseline),	T1CE
3 month follow up (third cycle of TMZ),	T2w
6 month follow up (sixth cycle of TMZ), and	T2-FLAIR
9 month follow up	APT (Must be available for 3 month follow up)
Survival Data	Overall survival at 12 months, OS-12.

Survival time was defined as the duration in days between the first follow-up after surgery and either patient death or the study’s conclusion. Patients who were alive at the time of outcome data collection were treated as censored observations, while deceased patients were treated as uncensored. All MRI studies were performed on an Ingenia 3.0T MR system with a 32-channel head coil (Philips Healthcare, Best, The Netherlands). APT data were acquired using the vendor-supplied 3D amide proton transfer (APT) imaging application, which automatically generates APT-weighted (APTw) images as used in this analysis. The APTw MRI acquisition protocol and scan parameters were kept consistent for all patients in the cohort. Key acquisition parameters are summarized in Table 2.

Imaging acquisition and data collection was undertaken under HREC approval from the Sir Charles Gairdner Osborne Park Health Care Group (RGS 5406), and from the University of Western Australia Human Research Ethics Office (2022/ET000602), with a waiver of consent.

**Table 2:** APTw MRI sequence parameters

	3D APTw sequence
FOV	230 × 179 × 60 mm^3^
Scan mode; Acquisition voxel; Reconstruction voxel	3D; 1.79 × 1.79 × 6.0 mm^3^; 0.89 × 0.89 × 6 mm^3^
Reconstruction matrix	256 × 256
Slice thickness, Slice gap	6 mm; 0 mm
SENSE or Compressed SENSE factor	1.6 SENSE
TSE factor	174
Rest slabs	0
Flip angle (in degrees)	90
TR, TE and TE equivalent	TR 6120 ms; TE 7.8 ms
Fat suppression	SPIR
APTw	saturation B1 rms (µT) = 2; saturation duration (s) = 2
Number of acquisitions NSA	1
Scan duration	3 min 46.4 s

**Fig. 1:**
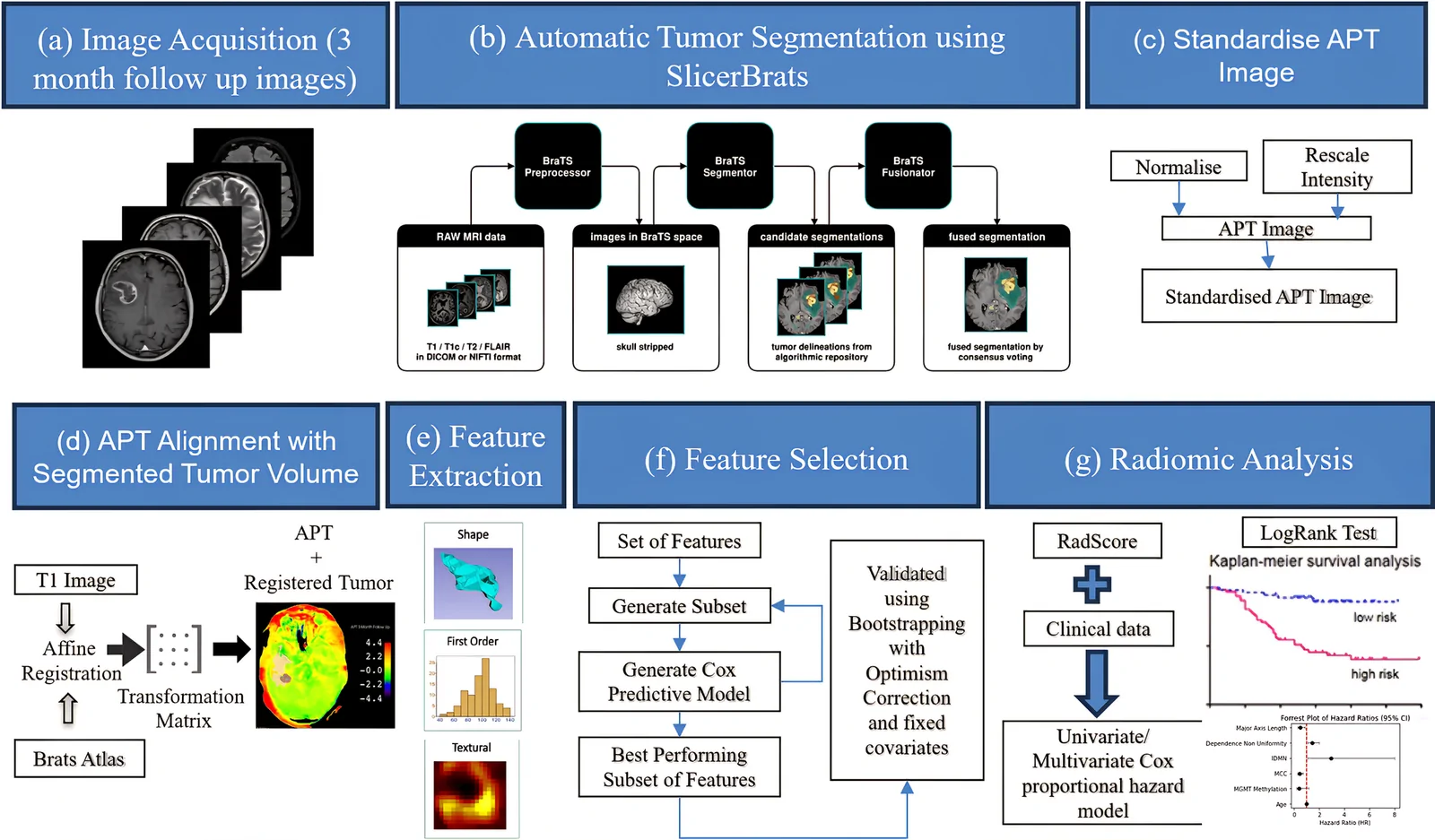
Workflow of radiomic process. **a** Image Acquisition, **b** Automatic Tumor Segmentation [18], **c** Standardise APT Image, **d** APT Registration to Tumor, **e** Feature Extraction, **f** Feature Selection and **g** Radiomic Analysis

### Automatic tumor segmentation

In this study, tumor volumes were delineated using deep learning-based segmentation through the BraTS toolkit [19, 20] (see Fig. 1b), employing models developed by Isensee et al. [21], McKinley et al. [22], Yuan [23] and Jia et al. [24]. The BraTS toolkit requires T1-weighted MRI with and without contrast enhancement, T2-weighted MRI, and FLAIR sequences. The segmentation results include: (a) the “active tumor,” defined by hyperintensity in contrast-enhanced T1-MRI compared to native T1-MRI and healthy white matter; (b) necrotic and non-enhancing tumor tissue; and (c) peritumoral edematous tissue visible in T2/FLAIR. The entire tumor volume, encompassing areas a–c, was used as the suspicious volume evident in FLAIR/T2 images, referred to as the “T2 volume”.

Segmentations generated by the BraTS Toolkit were validated against manually corrected segmentations by an experienced neuroradiologist for a subset of patients, using the Dice similarity score. The median values were 0.995 for necrosis, 0.998 for contrast-enhanced regions, and 0.972 for edema (see Table S1).

### Radiomic feature extraction

Radiomic features were extracted from each APT sub-region (a, b, c) after APT image standardization (see Fig. 1c). A SimpleITK-based pipeline normalized pixel intensities with sitk.Normalize and rescaled values to a range of -5 to 5 using sitk.RescaleIntensity, ensuring consistency for accurate feature extraction and analysis.

A custom 3D Slicer module using PyRadiomics (v3.0.1 [25]) was developed for radiomic feature extraction, following IBSI standards [26]. The module applied affine registration to align APT images with the BraTS reference space (see Fig. 1d), as tumor segmentations were defined in this space. Tumor segmentations were loaded, with specific regions sequentially made visible and exported as label maps. These label maps served as masks for radiomic feature extraction in PyRadiomics, enabling precise and standardized analysis of tumor regions. This workflow ensures accurate alignment and consistent radiomic data extraction across all patients. B-spline interpolation with a 0.1 bin width was used for resampling. The first feature set included 18 first-order statistics, and the second set included 75 texture features, including 14 Gy-level dependence matrix (GLDM), 24 Gy-level co-occurrence matrix (GLCM), 16 Gy-level run-length matrix, 16 Gy-level size zone matrix (GLSZM), and 5 neighboring gray-tone difference matrix (NGTDM) features. A total of 504 features were extracted for each patient, with no image filters applied.

### Single feature radiomics modelling

A Cox proportional hazard model [27, 28] was applied to identify relevant radiomic features for survival prediction based on calculated hazard ratios. The Cox regression was implemented using the lifelines package in python (version 0.29.0) [29]. Cox models were fitted to each radiomic features and potentially relevant features were identified based on the reported *p* value of the log-rank test. A 5% significance level was considered for hypothesis testing. The 95% confidence interval for the hazard ratio was computed based on the asymptotic distribution of the maximum likelihood estimator [28]. Univariate analysis was performed to assess the individual impact of each variable on survival outcomes.

### Feature reduction and multiple feature radiomics modelling

A two-step feature reduction process was applied: (1) Pearson correlation removed highly correlated features (correlation > 0.8), retaining 59 features by eliminating one from each correlated pair to reduce multicollinearity, (2) Due to censored data, cross-validation was not used. After removing highly correlated features, the best subset selection for Cox regression was applied using AIC, evaluating all feature combinations to balance model fit and complexity [30, 31]. The AIC served as the selection criterion, with the model exhibiting the lowest AIC value chosen. AIC penalizes models with excessive complexity, favouring simpler and more generalizable models. Minimizing the AIC identified a subset of four features that provided the best fit to the data while avoiding overfitting. For the features selected, we ran a goodness-of-fit test considering the likelihood ratio test, with the null hypothesis being all parameters (simultaneously) are equal to zero. Rejecting the null hypothesis provides evidence in favour of the selected features [28, 32]. To further assess the model performance, the C-index was computed [32]. Following the identification of the best subset of features, a bootstrapping [31, 33] with fixed covariates approach was applied to compute a 95% confidence interval for the C-index using 1000 resamples.

The radiomics model was developed with a multiple-feature cox proportional hazard model using the selected features. A Rad-score was generated via a linear combination of selected features weighted by their respective coefficients.

### Combination model construction

Three combination models were evaluated: one incorporating age, another including MGMT methylation, and a third combining both clinical parameters alongside the radiomic features.

### Kaplan Meier survival curves

Kaplan-Meier survival curves were generated using the lifelines library in Python, with “KaplanMeierFitter” for estimating survival probabilities and “add_at_risk_counts” for annotating plots. Statistical comparisons were performed using the “logrank_test” function. Data preprocessing was managed with “pandas”.

## Results

### Single feature radiomics modelling

When analysing the relevance of single radiomic features via the Cox proportional hazard model, several radiomic features from the edema region were associated (*p* < 0.05) with overall survival (see Table 2). Maximum Correlation Coefficient (MCC) (HR = 0.68, 95% CI 0.48–0.96, *p* = 0.028) and Informational Measure of Correlation 1 (IMC1) (HR = 1.91, 95% CI 1.07–3.42, *p* = 0.028) were the ones with small *p* values, with higher MCC values correlating with better survival outcomes, while higher IMC1 values were linked to worse survival. Additionally, the Surface Area to Volume Ratio (SVR) (HR = 0.63, *p* = 0.038) and Gray Level Non-Uniformity (GLN) (HR = 1.44, *p* = 0.042) from the edema region also had *p* value < 0.05. Overall, features from the edema region features emerged as survival predictors, with Table 2 showing those with *p* value < 0.05 in Cox proportional hazard models.

**Table 3:** Results of the radiomic features selected using the Cox Proportional Hazard Model

Feature^*^	HR^+^	*p* ^#^	C-index
MCC	0.677 (0.478, 0.959)	0.028	0.677
IMC1	1.912 (1.069, 3.419)	0.028	0.678
SVR	0.629 (0.406, 0.974)	0.038	0.644
GLN	1.440 (1.014, 2.046)	0.041	0.636

^*^MCC: Maximum Correlation Coefficient; IMC1: Informational Measure of Correlation 1; SVR: Surface Area to Volume Ratio; GLN: Gray Level Non-Uniformity

^+^HR: Hazard ratio and the 95% confidence interval in the rounded brackets

^#^*p* value for the hypothesis test of the parameter be equal to zero, which is equivalent to test the hypothesis of the hazard ratio being equal to 1. Rejecting the null hypothesis (*p* < 0.05) supports that the hazard ratio is different than 1

### Feature reduction and multiple feature radiomics modelling

In the final radiomic model, the selected features predominantly originate from the edema and necrosis regions of the tumour (see Table 3). Specifically, two features were identified from the edema region: the Maximal Correlation Coefficient (MCC) and the Inverse Difference Moment Normalized (IDMN), both derived from the GLCM. Additionally, one feature from the necrosis region, Dependence Non-Uniformity (DNU), was included, originating from GLDM. Lastly, a shape feature, Major Axis Length (MAL), was selected from the necrosis region.

The radiomic model, incorporating these four features (MCC, IDMN, DNU, MAL) yielded a c-index of 0.74, with a 95% confidence level of 0.666–0.852. The GOF test showed *p* < 0.005. The results are summarized in Table 3.

These four features were used to perform multiple feature analysis and all exhibited significant predictive ability, with *p* < 0.05 (log-likelihood ratio test). The features and their corresponding coefficients, along with their 95% CIs are shown in Table 3. This radiomic model consistently yielded a c-index of 0.74. The radiomic feature MAL, which was highlighted in a previous study [34] for its significant role in survival prediction, remains an important predictor in our current multiple feature model alongside other key features.

The rad-score, a linear combination of the selected image features weighted by their respective coefficients, was recorded for every patient [35]. The rad-score formula is: MCC ⋅ (− 0.65) + IDMN ⋅ (0.94) + DNU ⋅ (0.34) + MAL ⋅ (− 0.70).

**Table 4:** Hazard ratios of radiomic model

Features^*^	HR^+^	*p* ^#^
MCC (edema)	0.52 (0.36, 0.77)	< 0.005
IDMN (edema)	2.55 (0.99, 6.59)	0.05
DNU (necrosis)	1.41 (1.03, 1.93)	0.03
MAL (necrosis)	0.50 (0.28, 0.87)	0.01

^*^MCC: Maximum Correlation Coefficient; IDMN: Inverse Difference Moment Normalized (IDMN); DNU: Dependence Non-Uniformity; MAL: Major Axis Length (MAL).

^+^HR: Hazard ratio and the 95% confidence interval in the rounded brackets.

^#^*p* value for the hypothesis test of the parameter be equal to zero, which is equivalent to test the hypothesis of the hazard ratio being equal to 1. Rejecting the null hypothesis (*p* < 0.05) supports that the hazard ratio is different than 1.

MCC from the edema region showed a strong association, with higher values linked to a 57% reduced risk of death (HR = 0.52, 95% CI 0.36–0.77, *p* < 0.005). Similarly, the feature Gray-Level Co-occurrence Matrix Inverse Difference Moment (IDMN) from the edema region was associated with a higher risk of death (HR = 2.55, 95% CI 0.99–6.59, *p* = 0.05). The Gray-Level Dependence Matrix (GLDM) Dependence Non-Uniformity from the necrosis region modestly increased the risk (HR = 1.41, 95% CI 1.03–1.93, *p* = 0.03). The Major Axis Length shape feature from the necrosis region was associated with improved survival (HR = 0.50, 95% CI 0.28–0.87, *p* = 0.01).

### Combination model construction

The analysis was expanded by incorporating clinical parameters into the radiomic model. The impact of age alone, MGMT methylation alone, and both parameters combined with the radiomic features was evaluated to establish a comprehensive combination model. The results are presented in Table 5.

**Table 5:** Hazard ratios of combination models

Model Name	Features^*^	HR^+^	*p* ^#^
Radiomic model + Age	Age	0.99 (0.95, 1.03)	0.66
	MCC (edema)	0.52 (0.35, 0.76)	< 0.005
	IDMN (edema)	2.45 (0.94, 6.38)	0.07
	DNU (necrosis)	1.42 (1.03, 1.95)	0.03
	MAL (necrosis)	0.49 (0.28, 0.87)	0.01
Radiomic model + MGMT methylation	MGMT methylation	0.42 (0.15, 1.16)	0.0926
	MCC (edema)	0.43 (0.27, 0.71)	< 0.005
	IDMN (edema)	3.05 (1.15, 8.10)	0.01
	DNU (necrosis)	1.44 (1.05, 1.97)	0.02
	MAL (necrosis)	0.48 (0.27, 0.84)	0.01
Radiomics model + Age + MGMT methylation	Age	0.99 (0.95, 1.04)	0.66
	MGMT methylation	0.42 (0.15, 1.18)	0.11
	MCC (edema)	0.43 (0.26, 0.70)	< 0.005
	IDMN (edema)	2.97 (1.10, 8.01)	0.03
	DNU (necrosis)	1.45 (1.06, 1.99)	0.02
	MAL (necrosis)	0.48 (0.27, 0.84)	0.01

^*^MCC: Maximum Correlation Coefficient; IDMN: Inverse Difference Moment Normalized (IDMN); DNU: Dependence Non-Uniformity; MAL: Major Axis Length (MAL)

^+^HR: Hazard ratio and the 95% confidence interval in the rounded brackets

^#^*p* value for the hypothesis test of the parameter be equal to zero, which is equivalent to test the hypothesis of the hazard ratio being equal to 1. Rejecting the null hypothesis (*p* < 0.05) supports that the hazard ratio is different than 1

Considering the three models in Table 5, the hypothesis that the parameters for age and MGMT methylation features is not rejected (*p* > 0.05). We do not reject the hypothesis that their hazard ratios can equal 1. A hazard ratio close to 1 suggests that age had little to no impact on the risk of death when considered alongside the other variables in the model. This indicates that, for each additional year of age, the risk of death neither increased nor decreased significantly. However, MGMT methylation status yielded a hazard ratio of 0.42 (*p* = 0.0926) with confidence interval of 0.15–1.18. This finding suggests that MGMT methylation may be associated with a decreased risk of death in this cohort. When adding MGMT methylation as a clinical parameter to the model, the results indicate a slight decrease in performance compared to the radiomic-only model. The c-index was 0.74 (compared to 0.73 with MGMT methylation), with a 95% confidence interval of 0.672–0.864.

We also computed the AIC value and conducted the GOF test for all four models, with comparison results presented in Table 6. This test yielded significant *p* values for all models. Notably, the radiomic model combined with MGMT methylation demonstrated the best AIC value, indicating superior performance (lower values are better).

**Table 6:** Concordance Index, AIC Values, and *p* values (log-likelihood ratio test) for Radiomic and combination models

Model	c-index	AIC	*p*
Radiomics only model	0.74	191.35	< 0.005
Radiomics + Age	0.74	193.16	0.01
Radiomics + MGMT methylation	0.73	190.15	< 0.005
Radiomics + Age+MGMT methylation	0.73	192.08	< 0.005

From Table 6, all models have *p* < 0.05 for the GOF test, and their c-index are similar. The model with the lowest AIC was the Radiomics + MGMT methylation model, which we consider as our best model. The AIC measure penalises the addition of features to a model to avoid overfitting and keep a balance between model complexity and predictive capacity. Also, this supports the fact that MGMT methylation may be linked to a reduced risk of death.

### Kaplan Meier survival curves

**Fig. 2:**
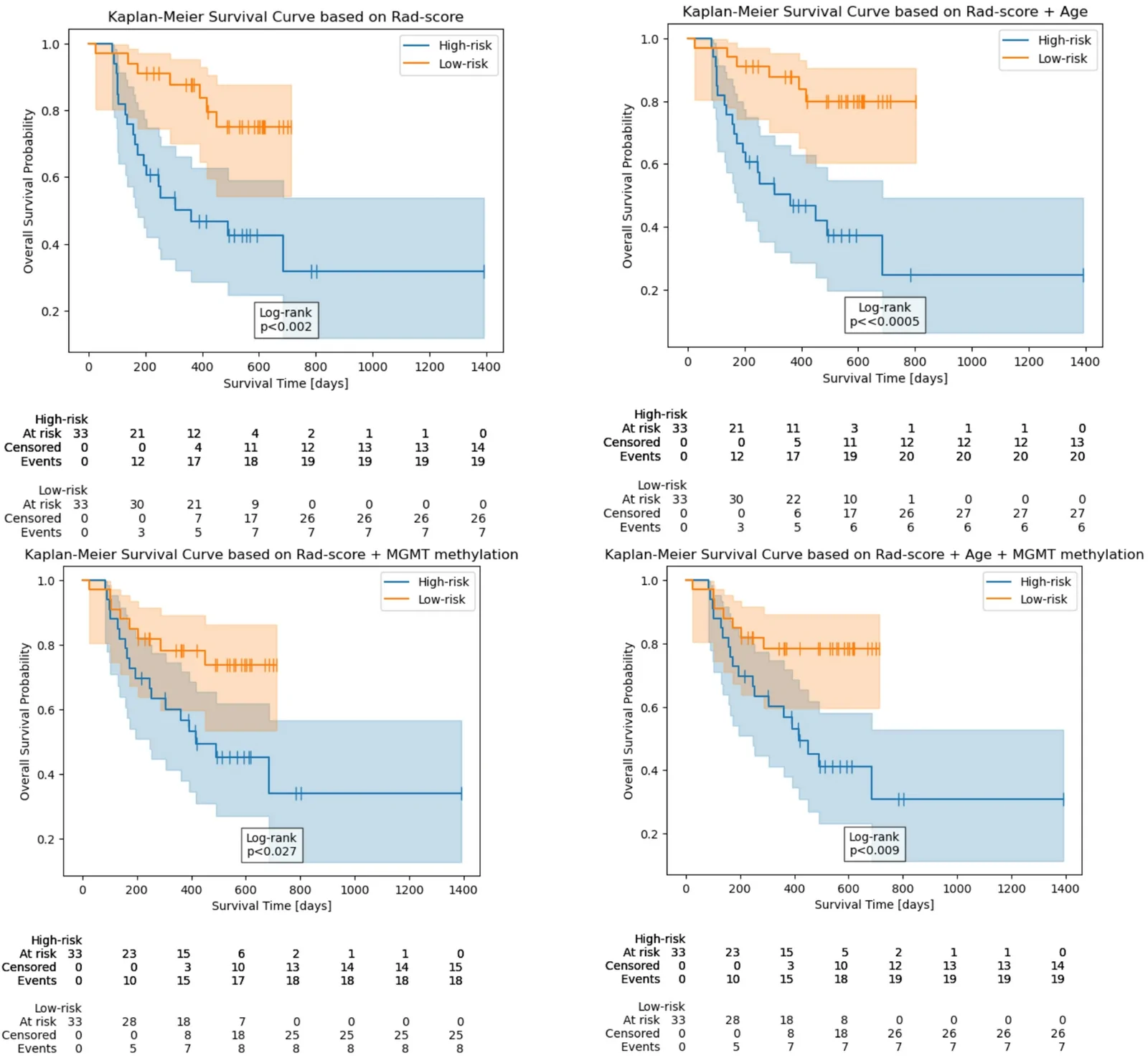
Kaplain-Meier Survival Curves for radiomic only model, radiomic + Age clinical parameter model, radiomic + MGMT methylation status clinical parameter model and radiomic + Age + MGMT methylation status model

The Kaplan-Meier survival curves presented in Fig. 2 illustrate how patients were stratified into high-risk and low-risk groups based on the radiomic score and other clinical factors, such as age and MGMT methylation status. The log-rank test comparisons show significant differences in survival probabilities between the risk groups across all four models achieving 95% statistical confidence, with *p* values consistently below 0.05.

## Discussion

In this study, radiomic features were extracted from APT images of glioblastoma patients using validated, semi-automated pipelines. Tumor segmentation was done with the BraTS toolkit, ensuring consistent feature extraction. The potential utility of extracted features was assessed by examining their correlation with patient overall survival. A two-step feature reduction minimized multicollinearity, and correlative radiomic features were selected using all subset Cox regression. The Bootstrap method evaluated the c-index confidence intervals. The feasibility of a model to predict overall survival was assessed. Incorporating clinical factors like MGMT methylation with radiomic features improved survival prediction.

When analysing the role of individual radiomic features, shape, GLCM and GLZSM features extracted from APT images were highlighted as the most relevant in the analysed dataset, especially the features from the edema region. Single feature Cox models were used to identify significant variables due to limitations associated with low event-per-variable ratios in Cox models, as discussed in [33]. Subsequently, a multiple feature analysis was conducted to evaluate the combined effects of multiple features on the survival outcome. When performing the best subset selection, the feature MCC from the edema region was also selected. The final model included features from shape, GLCM and GLSZM.

A closer analysis of the results reveals that higher values of the shape feature, Major Axis Length, representing the longest dimension of the necrotic region in post-surgical APT MRI images, are linked to better survival outcomes (HR = 0.48). This suggests that larger necrotic areas, which in part may correspond to the cavity left after tumor resection, are associated with less aggressive residual tumor behavior. The reduced risk tied to larger necrotic regions may indicate more effective tumor debulking, where less biologically active and more manageable tumor tissue remains post-surgery, potentially leading to improved patient prognosis [36].

In contrast, texture features from the edema region, such as GLCM MCC (Maximum Correlation Coefficient), continue to indicate a positive association with the patient outcome. Higher values of this feature, which reflect more uniform and structured tumor textures, are associated with a decrease in survival risk. This suggests that more organized and homogeneous tumor textures within the edema region are linked to improved survival outcomes (HR = 0.43, *p* < 0.005). One possible explanation is that more structured tumor tissue may indicate less aggressive tumor behavior, whereas disorganized or heterogeneous textures are often a hallmark of malignancy [37]. Tumors with more abnormal cellular architecture tend to behave more aggressively, growing and spreading at a faster rate. As such, the uniformity captured by higher GLCM MCC values may correspond to a less invasive tumor phenotype, contributing to a better prognosis for patients. Thus, the protective nature of this feature underscores the importance of texture homogeneity as a potential biomarker for improved survival in glioblastoma.

An in-depth analysis reveals that increasing values of GLCM IDMN (Inverse Difference Moment Normalized), which captures local homogeneity, are significantly associated with an elevated risk of mortality (HR = 3.05, *p* = 0.01). This suggests that a less uniform texture in the edema region, indicative of disorganization, is often linked to more aggressive tumor phenotypes and worse prognosis. The disrupted texture may reflect increased tumor infiltration and cellular irregularity, contributing to the heightened survival risk.

Similarly, GLSZM Dependence Non-Uniformity, measured in the necrosis region, shows that higher values, which indicate greater texture variation, are correlated with poorer survival outcomes (HR = 1.44, *p* = 0.02). This highlights the importance of tumor heterogeneity in driving aggressiveness, even in post-treatment APT MRI images taken three months after follow-up. Tumors with elevated texture variability in the necrosis region, reflected by higher Dependence Non-Uniformity, likely possess more complex and disorganized cellular structures. Despite surgical intervention, the persistence of varied textures in the necrotic region may indicate residual tumor activity or early regrowth, underscoring the prognostic significance of texture irregularity in necrotic regions as a marker of aggressiveness, even after treatment. When considering additional clinical factors, it was found that age was a non-significant predictor of survival in this cohort. However, the presence of MGMT methylation suggests [38] a marginally better survival outcome and contributes to enhancing the model’s predictive power, with its inclusion improving the AIC value.

A comparative analysis of our results with other studies is challenging due to the limited literature on the topic. amide proton transfer-weighted (APTw) imaging has been widely applied in the literature including tumor grading, prognostication, monitoring treatment response, and distinguishing tumor recurrence from treatment-related changes in glioma patients [8, 39, 40]. Previous studies have demonstrated its utility in predicting tumor grade, survival, and molecular profiles, such as IDH mutation status [13, 41]. In terms of radiomics, APTw imaging has shown promise in differentiating gliomas from metastases and classifying adult-type diffuse gliomas based on molecular and histopathological characteristics [16, 42]. However, we were unable to find studies that have specifically assessed the prognostic potential of radiomic features derived from APT images for predicting overall survival in glioblastoma patients.

This study has several limitations. It uses data from a single centre and a single Philips scanner, with each APT series limited to a maximum of 10 slices. This restricted coverage, along with potential scanner-specific artefacts, may have influenced the extracted radiomic features and reduced their generalisability. Although feature selection was performed using the best subset Cox model and uncertainty was assessed with bootstrapping, the absence of external validation means the predictive model requires confirmation in larger, multi-centre cohorts. The analysis also relied solely on APT imaging; incorporating additional MRI sequences may strengthen future radiomic models.

An absence of standardised APT processing workflows and inter-scanner harmonisation will limit translatability of results derived on this single-centre cohort, as previously identified [43, 44]. Due to likely limited harmonization of inter-scanner quantification, this study has focused on spatial features extracted from intensity-scaled and normalized image data. It is suggested that significant efforts are still required to enable generalisation of any APT MRI models based on quantitative metrics.

## Conclusion

This study demonstrated that radiomic features derived from APT images, in conjunction with clinical variables, correlate with overall survival in glioblastoma patients and show promise in predictive models. The best subset selection analysis identified key radiomic features, such as MCC and IDMN from the edema region and GLDM Dependence Non-Uniformity from the necrosis region. While age did not emerge as a significant predictor in this cohort, the inclusion of MGMT methylation status improved the model. These preliminary results should be extended to a larger patient cohort for further validation.

## Supplementary Information

Below is the link to the electronic supplementary material.

Supplementary Material 1

## Data Availability

Data remains unavailable due to privacy constraints.
