# Supplementary material for: Radiomic correlates with overall survival in glioblastoma: amide proton transfer (APT) imaging-based feature analysis and integration of clinical factors
Source: Phys Eng Sci Med. 2026 Jul 7;49(3):1459–68. doi: 10.1007/s13246-026-01757-9 (PMC13638680; doi:10.1007/s13246-026-01757-9)
Supplement: Supplementary file 1 [file 13246_2026_1757_MOESM1_ESM.docx]

| **Case_study** | **Necrosis (1)** | **Contrast_enhanced (2)** | **Edema (3)** |
| --- | --- | --- | --- |
| APT010 | 0.840241 | 0.992389 | 0.955464 |
| APT034 | 1 | 1 | 1 |
| APT038 | 0.9962 | 0.998311 | 0.970437 |
| APT045 | 0.9939 | 0.994193 | 0.974423 |
| APT049 | 0.999441 | 0.999661 | 0.951595 |
| APT055 | 0.992776 | 0.994589 | 0.98987 |
| APT057 | 0.988901 | 0.994521 | 0.899024 |
| APT058 | 1 | 1 | 0.989605 |
| APT059 | 1 | 1 | 0.990358 |
| APT072 | 1 | 1 | 1 |
| APT113 | 0.857762 | 0.973138 | 0.959709 |
| APT114 | 0.992714 | 0.988979 | 0.852493 |
| APT128 | 0.991467 | 0.987816 | 0.803209 |
| APT132 | 0.993664 | 0.997323 | 0.906489 |
| apt143 | 1 | 1 | 1 |
| apt154 | 1 | 1 | 1 |
| apt158 | 1 | 0.999676 | 0.981822 |
| apt172 | 1 | 1 | 1 |
| apt179 | 0.968817 | 0.989256 | 0.898927 |
| apt194 | 0.98387 | 0.986615 | 0.664606 |
| Median | 0.99505 | 0.997817 | 0.97243 |

Supplementary Table S1: Dice similarity score between Tumor Segmentations done automatically using Bratstoolkit and corrected segmentations by the experienced neuroradiologist.
